# Supplementary material for: Comparative genomics provides new insights into the diversity, physiology, and sexuality of the only industrially exploited tremellomycete: Phaffia rhodozyma
Source: BMC Genomics. 2016 Nov 9;17:901. doi: 10.1186/s12864-016-3244-7 (PMC5103461; doi:10.1186/s12864-016-3244-7)
Supplement: Additional file 6: — List of orphan genes with links to PFAM (related to Additional file 1: Table S1). (ZIP 1428 kb) [file 12864_2016_3244_MOESM6_ESM.zip › PFAM_UNIQUE/G05133_P.html]

#                                                                            --- full sequence --- -------------- this domain -------------   hmm coord   ali coord   env coord
  
# target name        accession   tlen query name           accession   qlen   E-value  score  bias   #  of  c-Evalue  i-Evalue  score  bias  from    to  from    to  from    to  acc description of target
  
#------------------- ---------- ----- -------------------- ---------- ----- --------- ------ ----- --- --- --------- --------- ------ ----- ----- ----- ----- ----- ----- ----- ---- ---------------------
  
G05133\_P             -            200 DUF566               PF04484.7    313     0.086   11.0   3.9   1   1   0.00038     0.098   10.8   3.9    20   117    45   164    31   194 0.59 - Family of unknown function (DUF566)  
G05133\_P             -            200 DUF605               PF04652.11   380       2.9    5.8  14.6   1   1     0.054       3.3    5.7  14.6   238   323    42   149    10   172 0.58 - Vta1 like  
G05133\_P             -            200 FA                   PF08736.6     47       2.6    6.6   5.3   1   3    0.0001      0.62    8.6   0.3    13    43    46    76    46    94 0.93 - FERM adjacent (FA)  
G05133\_P             -            200 FA                   PF08736.6     47       2.6    6.6   5.3   2   3      0.13   7.5e+02   -1.3   0.6    38    38   110   110    81   126 0.62 - FERM adjacent (FA)  
G05133\_P             -            200 FA                   PF08736.6     47       2.6    6.6   5.3   3   3      0.39   2.3e+03   -2.8   0.0    30    37   155   162   148   164 0.76 - FERM adjacent (FA)  
G05133\_P             -            200 TCRP1                PF14944.1    195     0.053   12.1   7.6   1   1   1.1e-05     0.066   11.8   7.6    30   116    60   146    37   176 0.84 - Tongue Cancer Chemotherapy Resistant Protein 1
